# Supplementary material for: A New Bacillus velezensis Strain CML532 Improves Chicken Growth Performance and Reduces Intestinal Clostridium perfringens Colonization
Source: Microorganisms. 2024 Apr 11;12(4):771. doi: 10.3390/microorganisms12040771 (PMC11051962; doi:10.3390/microorganisms12040771)
Supplement: Supplementary file 1 [file microorganisms-12-00771-s001.zip › microorganisms-2899699-supplementary.pdf]

Supplementary Materials

2. Materials and methods

2.1. Acid, bile salt and high temperature tolerance assay

For acid and bile salt tolerance assays, 0.1 mL of an overnight liquid culture of *Bacillu*s *velezensis* strains was inoculated into 10 mL of LB broth adjusted to pH 2 or 3 in advance, or into LB medium containing 0.3% or 0.4% (w/v) Oxgall (Beijing Solarbio Science & Technology Co., Ltd., Beijing, China) to imitate gastric juice at 37°C for 2 h. After incubation, the cell viability was determined by serial dilution and plating onto LB agar following a 12-hour incubation. Isolates that exhibit resistance above 80% at pH 3 are considered to be acid-tolerant strains. For temperature tolerance assay, 1 mL of *B. velezensis* liquid culture was immersed in a water bath at room temperature, 85°C and 90°C for 10 min, then cooled to room temperature, and incubated at 37°C for 12 h. Survival rate (%) = Final (Log CFU/mL)/Initial (Log CFU/mL) × 100%.

2.2. Protease assay

LB medium containing 1% skim milk powder was prepared, and a sterile hole punch with a diameter of 6 μm was used to drill holes in the medium. The supernatant of 50 μL *B. velezensi* was added to the well and cultured at 37°C for 24 h. Add 2 mL iodine solution and observe whether there is a transparent circle.

2.3. Amylase assay

LB medium containing 1% soluble starch (Sangon Biotech (Shanghai) Co., Ltd., Shanghai, China) was prepared, and a sterile hole punch with a diameter of 6 μm was used to drill holes in the medium. The supernatant of 50 μL *B. velezensis* was added to the well and cultured at 37°C for 24 h. Add 2 mL iodine solution and observe whether there is a transparent circle.

2.4. Cellulase assay

A sterilized hole punch with a diameter of 6 μm was used to punch holes in LB agar containing 1% carboxymethylcellulose sodium (Sangon Biotech (Shanghai) Co., Ltd., Shanghai, China), and then a sterile needle was used to pick out the drilled medium. The

supernatant of 50 μL *B. velezensis* fermentation broth was added to the well and cultured at 37°C for 24 h. Add 2 mL 0.2% congo red solution for 30 min, and replace 3 mL 1 M NaCl solution elute for 15 min, and observe whether there is a transparent circle.

2.5. Antibacterial Ability Test

The antimicrobial activity of *B. velezensis* against pathogenic bacteria *E. coli*, *S. typhimurium*, and *C. perfringens* was determined by the oxford cup method in accordance with Muhammad et al. [1]. Briefly, a 1% inoculum of pathogenic bacteria cultured overnight was plated onto LB agar. After the plate was dried, oxford cups were put onto the plate equidistantly with 30 μL test strains culture solution in each cup at 37°C for 24 h. The 100 μg/mL ampicillin and non-cultured MRS broth were used as positive and negative controls, respectively.

2.6. Antibiotic susceptibility assay

Antibiotic susceptibility was determined according to the Clinical and Laboratory Standards Institute (CLSI, 2010). The bacterial suspension was inoculated by swabbing on LB agar. After drying, the antibiotics discs were placed on the surface of the agar. The presence or absence of inhibition zones around the discs was recorded after incubation for 24 h at 37°C.

2.7. Intestinal lesion score

The small intestine of each chicken was cut longitudinally and scored blindly as described by Dahiya et al. [2] with some modifications**.** Lesions were assessed on a 0 to 4 scale. 0 = no gross lesions; 0.5 = severe congestive serous and mesenteric hyperemia; 1 = thin-walled, and brittle intestines with small hemorrhagic spots; 2 = small amount of gas production and focal necrotic lesions; 3 = gas-filled intestine and necrotic plaques (1–2 cm long); and 4 = large amount of gas and diffuse necrosis in the intestines.

References

1. Muhammad, Z.; Ramzan, R.; Abdelazez, A.; Amjad, A.; Afzaal, M.; Zhang, S. and Pan, S. Assessment of the antimicrobial potentiality and functionality of *lactobacillus plantarum* strains isolated from the conventional inner mongolian fermented cheese against foodborne pathogens. *Pathogens* **2019**, *8*, 71. https://doi.org/10.3390/pathogens8020071.
2. Dahiya, J. P.; Hoehler, D.; Wilkie, D. C.; Van Kessel, A. G. and Drew, M. D. Dietary

glycine concentration affects intestinal *clostridium perfringens* and *lactobacilli* populations in broiler chickens. *Poult. Sci.* **2005**, *84*, 1875-1885. https://doi.org/10.1093/ps/84.12.1875.

Table S1. Ingredients and nutrient composition of experimental diets used in experiment 1, as-fed basis.

**Ingredient, %**	**d 1–21**	**d 22–42**
Corn	56.05	59.87
Soybean meal (CP > 44%)	35.68	30.18
Soy oil	2.60	3.80
Corn gluten meal (CP > 51.3%)	2.00	2.90
Limestone	0.80	0.98
Dicalcium phosphate	1.90	1.41
Salt	0.30	0.30
Methionine (99%, DL-form)	0.22	0.10
Choline (50%)	0.20	0.20
Vitamin premix[1]	0.02	0.03
Mineral premix[2]	0.20	0.20
Ethoxyquin (66%)	0.03	0.03
B. velezensis premixc[3]	0.00	0.00
Total	100.00	100.00
Calculated composition (%)[4]		
Crude protein	21.54	20.00
Metabolizable energy (Kcal/kg)	2,970.00	3,095.00
Calcium	1.00	0.90
Available phosphorous	0.51	0.40
Lysine	1.21	1.07

[1] Provide per kilogram of vitamin premix: vitamin A, 50 million IU; vitamin D_3, 12 million IU; vitamin E, 0.1 million IU; vitamin K_3, 10 g; vitamin B_1, 8 g; vitamin B_2, 32 g; vitamin B_6, 12 g; vitamin B_{12}, 100 mg; nicotinamide, 150 g: D-pantothenic acid, 46 g; folic acid, 5 g: biotin, 500 mg

[2] Provide per kilogram of mineral premix: copper, 8 g: iron, 40 g; zinc,55 g: manganese, 60 g; iodine, 750 mg: selenium, 150 mg: cobalt, 250 mg.

[3] *Bacillus velezensis* premix was added at the expense of corn to supply 0 or 1×10^9 CFU/kg diet

[4] Calculated values based on the analyzed data for the experimental diets

Table S2. Ingredients and nutrient composition of experimental diets used in experiment 2, as-fed basis.

**Ingredient, %**	**d 1–21**	**d 22–42**
Corn	56.77	59.87
Soybean meal (CP > 44%)	34.11	30.18
Soy oil	2.64	3.80
Corn gluten meal (CP > 51.3%)	2.5	2.90
Limestone	0.88	0.98
Dicalcium phosphate	1.89	1.41
Salt	0.35	0.30
Methionine (99%, DL-form)	0.19	0.10
L-lysinehydrochloride (78%)	0.11	0.14
Choline (50%)	0.30	0.20
Vitamin premix[1]	0.03	0.03
Mineral premix[2]	0.20	0.20
Ethoxyquin (66%)	0.03	0.03
B. velezensis premixc[3]	0.00	0.00
Total	100.00	100.00
Calculated composition (%)[4]		
Crude protein	21.54	20.00
Metabolizable energy (Kcal/kg)	2,970	3,100
Calcium	1.00	0.90
Available phosphorous	0.51	0.40
Lysine	1.21	1.07

[1] Provide per kilogram of vitamin premix: vitamin A, 50 million IU; vitamin D_3, 12 million IU; vitamin E, 0.1 million IU; vitamin K_3, 10 g; vitamin B_1, 8 g; vitamin B_2, 32 g; vitamin B_6, 12 g; vitamin B_{12}, 100 mg; nicotinamide, 150 g: D-pantothenic acid, 46 g; folic acid, 5 g: biotin, 500 mg

[2] Provide per kilogram of mineral premix: copper, 8 g: iron, 40 g; zinc,55 g: manganese, 60 g; iodine, 750 mg: selenium, 150 mg: cobalt, 250 mg.

[3] *Bacillus velezensis* premix was added at the expense of corn to supply 0 or 1×10^9 CFU/kg diet

[4] Calculated values based on the analyzed data for the experimental diets

Table S3. Forward and reverse primers for quantitative PCR.

Gene	Accession no.	Primer sequence (5′–3′) Accession no.
β-actin	L08165	F：GAGAAATTGTGCGTGACATCA<br>R：CCTGAACCTCTCATTGCCA
Occludin	GI:464148	F：ACGGCAGCACCTACCTCAA<br>R：GGGCGAAGAAGCAGATGAG
ZO-1	XM_413773	F：CTTCAGGTGTTTCTCTTCCTCCTC<br>R：CTGTGGTTTCATGGCTGGATC
Claudin-2	NM_001277622.1	F：CTGCTCACCCTCATTGGA<br>R：AACTCACTCTTGGGCTTCTG
Claudin-3	NM_204202.2	F：CCAAGATCACCATCGTCTCC<br>R：CACCAGCGGGTTGTAGAAAT
Mucin-2	XM_421035	F：TTCATGATGCCTGCTCTTGTG<br>R：CCTGAGCCTTGGTACATTCTTGT
IL-1β	NM_204524	F：ACTGGGCATCAAGGGCTA<br>R：GGTAGAAGATGAAGCGGGTC
IL-6	AJ250838	F：GCTCGCCGGCTTCGA<br>R：GGTAGGTCTGAAAGGCGAACAG
IL-8	NC_006091.5	F：GGTCTCGGAGCAGCGGATAG<br>R ：GTCTCTGTGATGGAGCCATTCATG
IL-17	NM_204460.1	F：TATCAGCAAACGCTCACTGG<br>R：AGTTCACGCACCTGGAATG
TNF-α	NM_204267	F：GAGCGTTGACTTGGCTGTC<br>R：AAGCAACAACCAGCTATGCAC
IFN-γ	NM_205149.1	F：AGCTGACGGTGGACCTATTATT<br>R：GGCTTTGCGCTGGATTC
GLUT1	NM_205209.1	F：TCCTCCTGATCAACCGCAAT<br>R：TGTGCCCCGGAGCTTCT
GLUT2	Z22932	F：CACACTATGGGCGCATGCT<br>R：ATTGTGCCTGGAGGTGTTGGT
PepT1	NM_204365.1	F：CCCCTGAGGAGGATCACTGTT<br>R：CAAAAGAGCAGCAGCAACGA
SGLT1	NM_001293240	F：GCCATGGCCAGGGCTTA<br>R：CAATAACCTGATCTGTGCACCAGTA
T1R1	XM_425734.4	F：GTGTCATCCCCACAACCAA<br>R：CACCACTGCCTCAAAGAAGG
T1R3	XM_425740.3	F：CATTACCGTCTTCGCCACTC<br>R：CTCTGTTCAAATCGGGCTTC

β -Actin = Beta-actin; *IL-1β* = interleukin 1 beta; *IL-6* = interleukin 6; *IL-17* = interleukin 17; *NF-kB* = nuclear factor kappa B; *TNF-α* = tumor necrosis factor alpha; IFN-γ = interferon gamma; *GLUT1* = glucose transporter 1; *GLUT2* = glucose transporter 2; *PepT1* = Peptide transporter-1; *SGLT1* = sodium-glucose transporter 1; *T1R1* = taste receptor type 1 member 1; *T1R3* = taste receptor type 1 member 3.

Table S4. Information of *Bacillus* isolates from the chicken gut.

ID	Phylum	Genus	Species	Chicken Breed	Origin
			Bacillus altitudinis		
CML488	Firmicutes	*Bacillus*	*Bacillus altitudinis*	Beijing fatty chicken	Beijing
CML496	Firmicutes	*Bacillus*	*Bacillus altitudinis*	817 chicken	Yunfu, Guangdong
CML500	Firmicutes	*Bacillus*	*Bacillus altitudinis*	Mahuanggong chicken	Yunfu, Guangdong
CML515	Firmicutes	*Bacillus*	*Bacillus altitudinis*	Aixiang chicken	Yunfu, Guangdong
CML522	Firmicutes	*Bacillus*	*Bacillus altitudinis*	Tuer chicken	Yunfu, Guangdong
CML525	Firmicutes	*Bacillus*	*Bacillus altitudinis*	Shanzhongxian hen	Xuancheng, Anhui
CML536	Firmicutes	*Bacillus*	*Bacillus altitudinis*	Shanzhongxian rooster	Xuancheng, Anhui
CML540	Firmicutes	*Bacillus*	*Bacillus altitudinis*	Shiqi chicken	Jinan, Shandong
CML544	Firmicutes	*Bacillus*	*Bacillus altitudinis*	Luhua chicken	Jinan, Shandong
			Bacillus atrophaeus		
CML474	Firmicutes	*Bacillus*	*Bacillus atrophaeus*	Nongda third chicken	Beijing
CML527	Firmicutes	*Bacillus*	*Bacillus atrophaeus*	Shanzhongxian hen	Xuancheng, Anhui
			Bacillus cereus		
CML481	Firmicutes	*Bacillus*	*Bacillus cereus*	Nongda third chicken	Beijing
CML484	Firmicutes	*Bacillus*	*Bacillus cereus*	Green shell layer	Beijing
CML506	Firmicutes	*Bacillus*	*Bacillus cereus*	Jianghan chicken	Wuhan, Hubei
CML533	Firmicutes	*Bacillus*	*Bacillus cereus*	Shanzhongxian rooster	Xuancheng, Anhui
			Bacillus licheniformis		
CML477	Firmicutes	*Bacillus*	*Bacillus licheniformis*	Nongda third chicken	Beijing
CML490	Firmicutes	*Bacillus*	*Bacillus licheniformis*	Beijing fatty chicken	Beijing
CML493	Firmicutes	*Bacillus*	*Bacillus licheniformis*	817 chicken	Yunfu, Guangdong
CML524	Firmicutes	*Bacillus*	*Bacillus licheniformis*	Tuer chicken	Yunfu, Guangdong
			Bacillus paralicheniformis		
CML502	Firmicutes	*Bacillus*	*Bacillus paralicheniformis*	Jianghan chicken	Wuhan, Hubei
CML514	Firmicutes	*Bacillus*	*Bacillus paralicheniformis*	Aixiang chicken	Yunfu, Guangdong
CML519	Firmicutes	*Bacillus*	*Bacillus paralicheniformis*	Yuqingxiang chicken	Yunfu, Guangdong
CML521	Firmicutes	*Bacillus*	*Bacillus paralicheniformis*	Tuer chicken	Yunfu, Guangdong
CML538	Firmicutes	*Bacillus*	*Bacillus paralicheniformis*	Bairi chicken	Jinan, Shandong
CML541	Firmicutes	*Bacillus*	*Bacillus paralicheniformis*	Shiqi chicken	Jinan, Shandong
CML543	Firmicutes	*Bacillus*	*Bacillus paralicheniformis*	Luhua chicken	Wenshang, Shandong
CML547	Firmicutes	*Bacillus*	*Bacillus paralicheniformis*	Langya chicken	Langya, Shandong
			Bacillus siamensis		
CML475	Firmicutes	*Bacillus*	*Bacillus siamensis*	Nongda third chicken	Beijing
CML492	Firmicutes	*Bacillus*	*Bacillus siamensis*	817 chicken	Yunfu, Guangdong
CML499	Firmicutes	*Bacillus*	*Bacillus siamensis*	Mahuanggong chicken	Yunfu, Guangdong
CML503	Firmicutes	*Bacillus*	*Bacillus siamensis*	Jianghan chicken	Wuhan, Hubei
CML507	Firmicutes	*Bacillus*	*Bacillus siamensis*	Lueyang chicken	Lueyang,Shanxi
CML513	Firmicutes	*Bacillus*	*Bacillus siamensis*	Aixiang chicken	Yunfu, Guangdong
CML517	Firmicutes	*Bacillus*	*Bacillus siamensis*	Yuqingxiang chicken	Yunfu, Guangdong
			Bacillus tequilensis		
CML479	Firmicutes	*Bacillus*	*Bacillus tequilensis*	Nongda third chicken	Beijing
CML489	Firmicutes	*Bacillus*	*Bacillus tequilensis*	Beijing fatty chicken	Beijing
CML491	Firmicutes	*Bacillus*	*Bacillus tequilensis*	817 chicken	Yunfu, Guangdong
CML504	Firmicutes	*Bacillus*	*Bacillus tequilensis*	Jianghan chicken	Wuhan, Hubei
CML518	Firmicutes	*Bacillus*	*Bacillus tequilensis*	Yuqingxiang chicken	Yunfu, Guangdong
CML520	Firmicutes	*Bacillus*	*Bacillus tequilensis*	Tuer chicken	Yunfu, Guangdong
CML528	Firmicutes	*Bacillus*	*Bacillus tequilensis*	Shanzhongxian hen	Xuancheng, Anhui

CML534	Firmicutes	*Bacillus*	*Bacillus tequilensis*	Shanzhongxian rooster	Xuancheng, Anhui
			Bacillus velezensis		
CML532	Firmicutes	*Bacillus*	*Bacillus velezensis*	Shanzhongxian rooster	Xuancheng, Anhui
CML526	Firmicutes	*Bacillus*	*Bacillus velezensis*	Shanzhongxian hen	Xuancheng, Anhui
CML537	Firmicutes	*Bacillus*	*Bacillus velezensis*	Bairi chicken	Jinan, Shandong
CML539	Firmicutes	*Bacillus*	*Bacillus velezensis*	Shiqi chicken	Jinan, Shandong
CML542	Firmicutes	*Bacillus*	*Bacillus velezensis*	Luhua chicken	Jinan, Shandong
CML546	Firmicutes	*Bacillus*	*Bacillus velezensis*	Langya chicken	Langya, Shandong
			**Others**		
CML476	Firmicutes	*Bacillus*	*Bacillus halotolerans*	Nongda third chicken	Beijing
CML478	Firmicutes	*Bacillus*	*Bacillus aryabhattai*	Nongda third chicken	Beijing
CML482	Firmicutes	*Bacillus*	*Bacillus bingmayongensis*	Nongda third chicken	Beijing
CML483	Firmicutes	*Bacillus*	*Bacillus circulans*	Nongda third chicken	Beijing
CML494	Firmicutes	*Bacillus*	*Bacillus oleronius*	817 chicken	Yunfu, Guangdong
CML495	Firmicutes	*Bacillus*	*Bacillus sonorensis*	817 chicken	Yunfu, Guangdong
CML497	Firmicutes	*Bacillus*	*Bacillus fordii*	817 chicken	Yunfu, Guangdong
CML501	Firmicutes	*Bacillus*	*LDWH_s*	Mahuanggong chicken	Yunfu, Guangdong
CML505	Firmicutes	*Bacillus*	*Bacillus subtilis*	Jianghan chicken	Wuhan, Hubei
CML510	Firmicutes	*Bacillus*	*Bacillus marisflavi*	Lueyang chicken	Lueyang,Shanxi
CML529	Firmicutes	*Bacillus*	*Bacillus wiedmannii*	Shanzhongxian hen	Xuancheng, Anhui
CML535	Firmicutes	*Bacillus*	*Bacillus hisashii*	Shanzhongxian rooster	Xuancheng, Anhui

Table S5. The annotation of resistance genes in *B. velezensis* CML532 genome.

Resistance genes	Identity, %	Coverage rate, %	Start	Stop
Cfr(B)	89.09	98.67	570,335	571,384
tet(L)	86.86	100.00	2,587,728	2,589,104

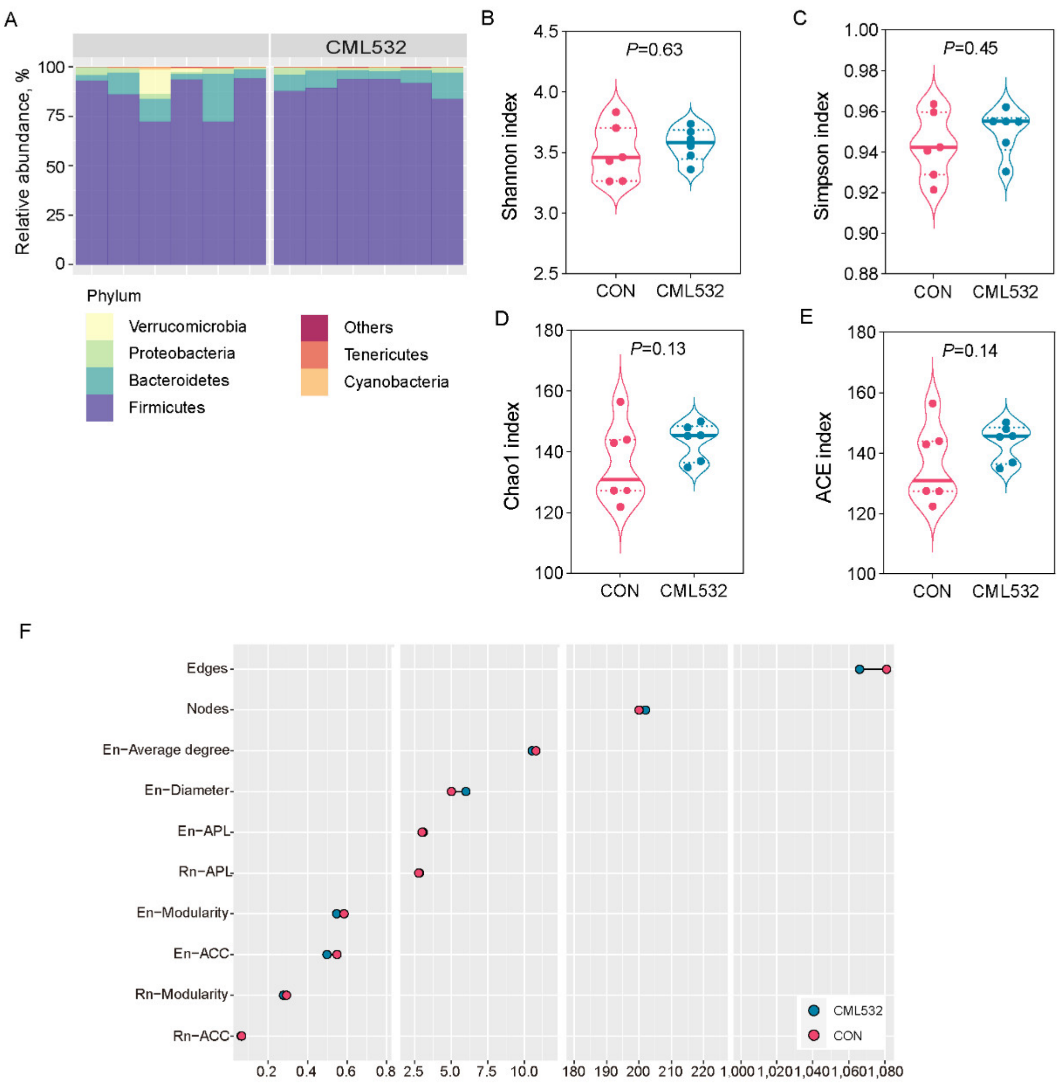

Figure S1. *B. velezensis* CML532 changes the cecal microbial community structure. (**A**) Relative abundance of major bacterial phylum. (**B–E**) Microbiome α-diversity in *B. velezensis* CML532 and control groups at genus-level. (**F**) Empirical and randomized molecular ecology network properties of microbial communities under different treatment. Randomized networks were performed by rewiring all the nodes and links corresponding to empirical networks 1,000 times. En: Empirical network. Rn: Randomized networks. ACC: Average clustering coefficient. APL: Average path distance.
